# Feasibility of the Pleural Sliding Test for Detection and Management of Lung Adhesions During Robotic Cardiac Surgery

**DOI:** 10.3390/jcdd13100495

**Published:** 2026-10-01

**Authors:** Murat Bastopcu, Gökhan Arslanhan, Zeynep Sıla Özcan, Anıl Karaağaç, Muharrem Koçyiğit, Halim Ulugöl, Şahin Şenay, Cem Alhan

**Affiliations:** 1 Department of Cardiovascular Surgery, Faculty of Medicine, Acıbadem University Mehmet Ali Aydınlar, 34638 Istanbul, Turkey; murat.bastopcu@acibadem.edu.tr (M.B.);; 2 Department of Cardiovascular Surgery, Acıbadem Altunizade Hospital, 34662 Istanbul, Turkey; 3 Department of Anesthesiology, Acıbadem Maslak Hospital, 34457 Istanbul, Turkey; 4 Department of Anesthesiology, Acıbadem Altunizade Hospital, 34662 Istanbul, Turkey

**Keywords:** robotic-assisted surgery, robotic cardiac surgery, ultrasonography, pleural sliding test, perioperative care

## Abstract

Pleural adhesions can complicate access in robotic cardiac surgery, risking lung injury or conversion to sternotomy. The sliding test using thoracic ultrasound can predict thoracic adhesions and guide the surgeon during robotic set-up. We evaluated the utility of the sliding test in robotic cardiac surgery. The pleural sliding test was performed in 300 consecutive robotic cardiac surgery patients between August 2022 and September 2025. The test was performed by the surgeon using a linear probe at six intercostal sites after intubation. Intraoperative findings were used to validate the test. Tailored port placement and minithoracotomy strategies were used when adhesions were suspected. The mean age was 60.3 ± 13.7 years and 91 (30.3%) of the patients were female. Adhesions were encountered in 20 (6.7%) patients. Prior cardiothoracic surgery was associated with thoracic adhesions (*p* < 0.001). The pleural sliding test had a sensitivity of 95.0%, specificity of 97.9%, positive predictive value of 76.0%, negative predictive value of 99.6%. No pulmonary injuries occurred and no conversions to sternotomy were necessary in the cohort. Patients with adhesions had higher 24 h chest tube output (*p* = 0.006) but had similar mechanical ventilation times and intensive care unit stay durations. Pleural adhesions are not rare in patients undergoing robotic cardiac surgery, and their optimal management enhances the safety and efficiency of robotic cardiac procedures. The pleural sliding test offers high accuracy for preoperative detection, enabling tailored access strategies that enhance safety and maintain benefits of the robotic approach.

## 1. Introduction

Robotic-assisted surgery offers the most minimally invasive approach to cardiac operations with reduced operative trauma and quicker recovery compared to traditional sternotomy approaches. The robotic approach in cardiac surgery is through 8 mm ports in the intercostal spaces or a minithoracotomy for the insertion of robotic camera and arms. Pleural adhesions in cardiac surgery patients associated with prior thoracotomy, pneumonia, thoracic drains, or radiotherapy can prevent lung collapse and complicate thoracic access in robotic cardiac surgery [1,2]. The risk of pulmonary injury during port placement is increased and extensive, dense adhesions may limit the working space of the surgeon, increasing operative time or necessitating conversion to more invasive approaches [3].

Transthoracic ultrasound offers a non-invasive method to detect pleural adhesions by assessing the dynamic movement of the visceral pleura relative to the parietal pleura during respiration. Restricted sliding or absence of the “gliding sign” indicates potential adhesions, a principle well-established in diagnosing conditions like pneumothorax and increasingly applied in preoperative thoracic evaluations. Studies have demonstrated its utility in predicting pleural adhesions prior to thoracic surgery, with high sensitivity, positioning it as an effective rule-in test to guide port placement and adhesiolysis strategies [4,5,6,7].

We investigated the incidence of pleural adhesions in a cohort of robotic cardiac surgery patients and evaluated the utility of the preoperative pleural sliding test in the management of thoracic adhesions.

## 2. Materials and Methods

The study included 300 consecutive robotic cardiac surgery patients operated between August 2022 and September 2025 in a single center experienced in robotic cardiac surgery. All patients were evaluated with a detailed patient history and preoperative thoracoabdominal contrast enhanced computed tomography. No patient with prior right sided thoracotomy was excluded from robotic surgery. Patient characteristics including demographics, comorbidities, previous cardiothoracic surgery, and operative outcomes were prospectively recorded. The study was conducted in accordance with the Declaration of Helsinki and approved by the Institutional Review Board. Written informed consent has been obtained from the study patients.

### 2.1. The Pleural Sliding Test

**Figure 1:**
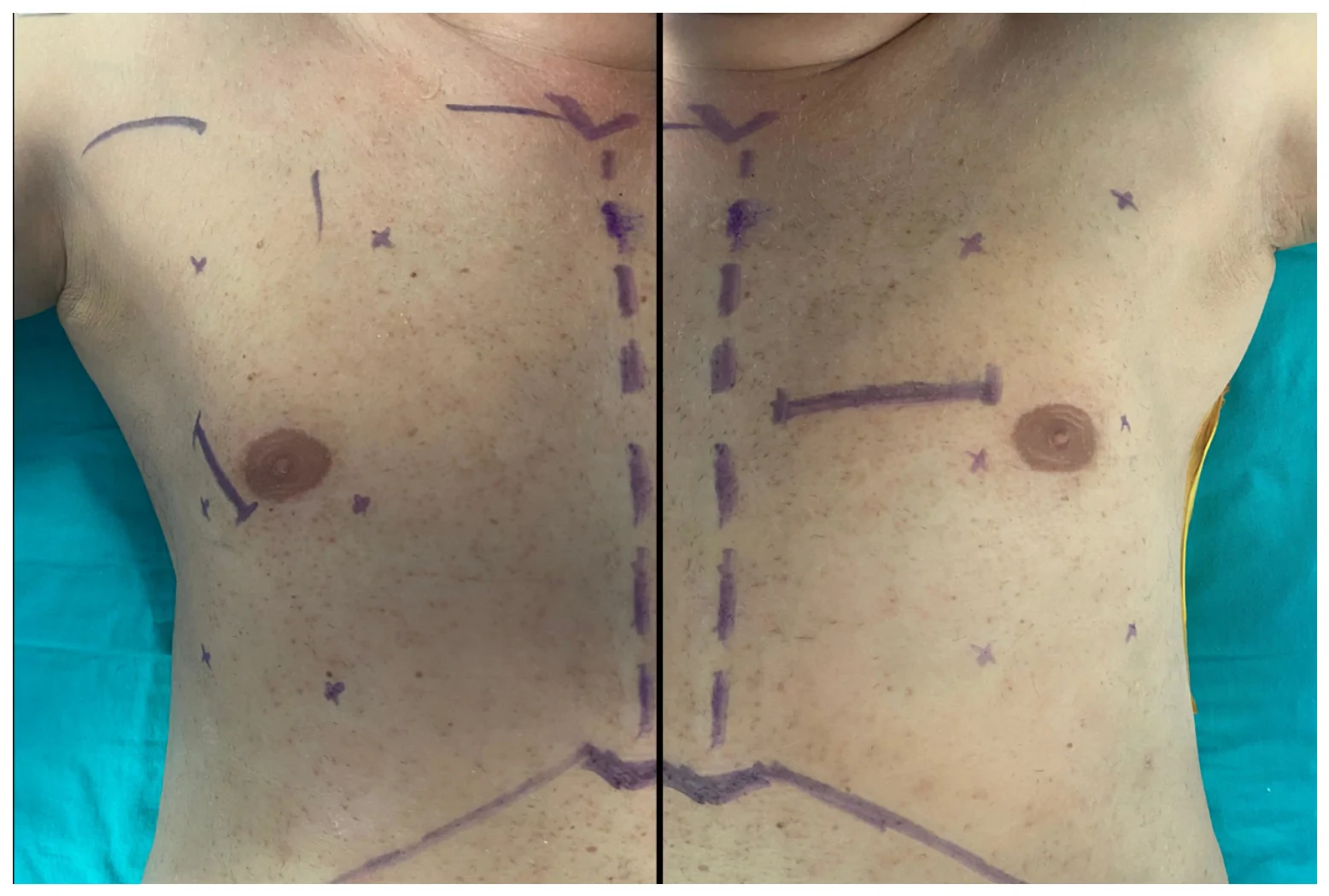
The sliding test is performed at six locations on the surgical side: the 2nd, 4th, and 6th intercostal spaces along the midclavicular and anterior axillary lines.

**Figure 2:**
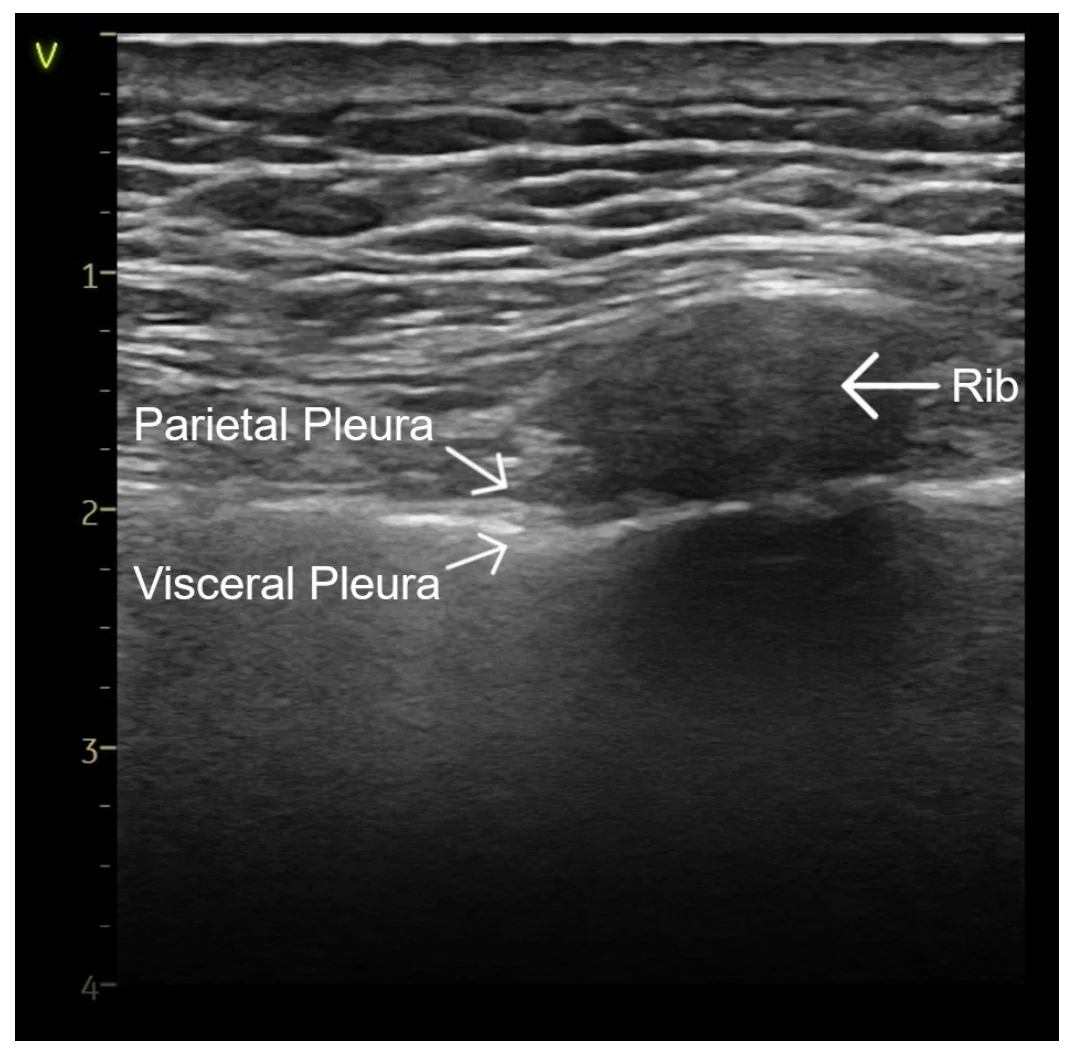
The parietal and visceral pleura are visualized with high-frequency ultrasound.

Thoracic ultrasound was performed by the same senior surgeon in the operating theater after intubation with the patient in the supine position. An 11L-D linear probe (VIVID S60, GE Healthcare, Chicago, IL, USA) at B-mode was used to image the target thoracic side at six locations: the 2nd, 4th, 6th intercostal spaces along the midclavicular and anterior axillary lines (Figure 1). The movement of the visceral pleura during double lung ventilation was assessed (Figure 2). Restricted sliding (<1 cm in the upper thoracic wall or <2 cm in the middle or lower thoracic wall) of the selected point in the intercostal space or pulling of the intercostal muscle during respiration was indicative of potential adhesions [8,9]. The sliding test at six designated locations was recorded and compared with operative findings to assess the predictive ability of the test (Supplementary Video S1).

### 2.2. Tailored Approach

**Figure 3:**
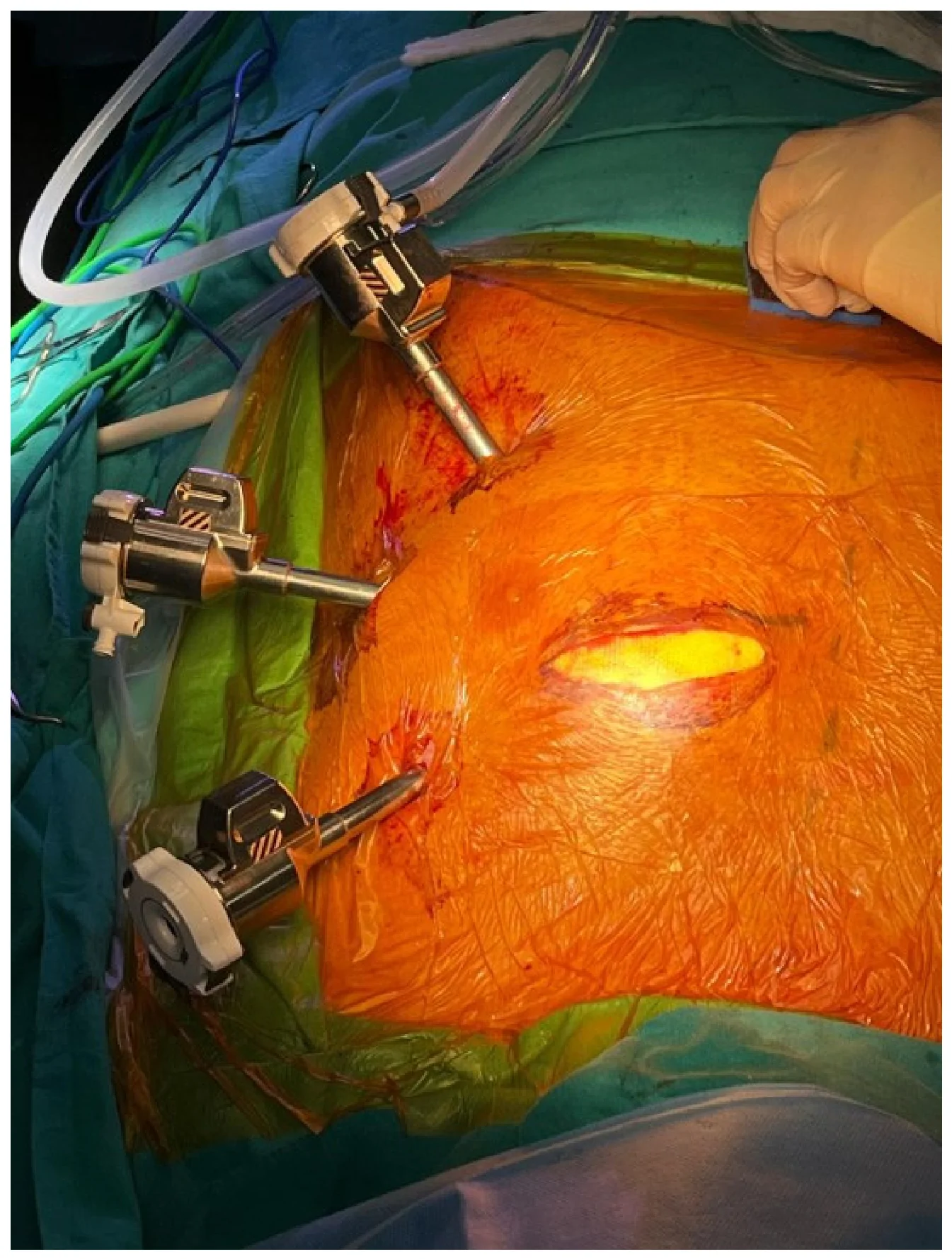
The minithoracotomy can be performed before any port placement in cases of expected adhesions at all port entry sites.

Our standard techniques for robotic mitral and robotic-assisted minimally invasive direct coronary artery bypass (MIDCAB) have been detailed elsewhere [10,11]. In robotic-assisted MIDCAB cases, if no adhesions were suspected, the port placement was carried out per routine. If adhesions were suspected in the camera port area, the initial port was placed in a quadrant with no adhesions, and subsequent ports were placed under camera guidance. If all port sites showed adhesions, a minithoracotomy was performed for adhesiolysis before port placement (Figure 3). After safe port placement, the minithoracotomy is draped to seal the thoracic cavity for carbondioxide insufflation during robotic internal mammary artery harvest.

For valve and other surgeries via the right thorax, when adhesions were suspected, the minithoracotomy was performed cautiously. The sliding test was used to guide the site of the anterolateral minithoracotomy and port entry in the thoracic wall where adhesions were less suspected. A small space was created with careful electrocautery of the pleura and peanut gauze or gentle finger dissection. Endoscopic vision through the thoracoscopy was used to assess the extent of adhesions, plan port entry sites, and guide port insertion. In severe adhesions, the port sites were adjusted on intercostal space above or below as adhesions permitted. After completion of port insertions, adhesiolysis continued with the robotic instruments. A working space was created in the thoracic cavity just enough to reach the pericardium of the atria. The operation was performed on pump on the beating heart or fibrillating heart if adhesions prohibited exposure of the aorta.

### 2.3. Statistical Analysis

Statistical analysis was carried out using IBM SPSS Statistics version 25.0 (IBM, Corp., Armonk, NY, USA) and R (version 4.3.1, R Foundation for Statistical Computing, Vienna, Austria). Continuous parameters are given as mean and standard deviation, while categorical parameters are given as numbers and percentages. The Shapiro–Wilk test was used to assess normal distribution. The chi-squared test was applied to compare categorical variables. Student’s *t* test was used for comparisons between 2 groups of normally distributed continuous variables, and Mann–Whitney U test was used for comparisons between 2 groups of non-normally distributed continuous variables. Confidence intervals for sensitivity, specificity, and predictive values were calculated using Clopper–Pearson Exact method.

## 3. Results

The mean patient age was 60.3 ± 13.7 years, and 91 (30.3%) were female. Baseline characteristics of the study patients are given in Table 1. 16 (5.3%) patients had a history of prior cardiac surgery and 5 (1.7%) had a prior ipsilateral thoracotomy, with one patient having both. Cases included 163 (54.3%) robotic-assisted MIDCAB, 118 (39.3%) valve procedures, and 19 (6.3%) other procedures (atrial septal defect repair, myxoma resection, partial anomalous pulmonary venous return repair, atrial thrombus excision, and pericardial window). The types of operations of the study patients are detailed in Table 2.

Adhesions were identified during surgery in 20 (6.7%) patients. Demographic characteristics of patients with and without adhesions were similar. Notably, previous cardiac or ipsilateral thoracic surgery was more frequent (65.0% vs. 1.1%, *p* < 0.001) in patients with adhesions. Cardiopulmonary bypass and cross-clamp durations were not longer in patients with adhesions. The duration of mechanical ventilation (6.1 ± 15.4 vs. 8.1 ± 8.4 h, *p* = 0.148) and intensive care unit stay (24.2 ± 22.3 vs. 33.9 ± 24.9 h, *p* = 0.102) were similar between patients with and without adhesions. 24 h chest tube output was higher (1100.2 ± 1025.4 vs. 533.7 ± 426.5 mL, *p* = 0.006) and blood transfusions were more frequently required (30.0% vs. 6.8%, *p* = 0.003) in patients with adhesions. One (5.0%) patient with adhesions required a reoperation for bleeding. No cases of pulmonary injury were observed and no patient required conversion to sternotomy (Table 3).

The pleural sliding test showed high diagnostic accuracy in the detection of pleural adhesions (Table 4). In our cohort of robotic cardiac operations, the test had a sensitivity of 95.0% and a specificity of 97.9%. The sliding test was able to rule out adhesions in a high majority of cases with a negative predictive value of 99.6%. The positive predictive value of the test was 76.0%, with 6 false-positive test results.

Among the 16 patients with previous cardiothoracic surgery, 13 (81.3%) had intraoperative adhesions. The sliding test was positive in 12 cases, and one patient had a false-negative test. Among the 284 patients without previous surgery, adhesions were encountered in 7 (2.5%) cases. The test detected all 7 patients with adhesions, and 6 patients had false-positive results in this group (Table 5). Of these 7 patients with adhesions, one had a history of pneumonia and thoracic radiotherapy. The remaining 6 had no identifiable risk factor for pleural adhesions.

## 4. Discussion

Robotic approach offers advantages in cardiac operations when compared to sternotomy or other minimally invasive approaches, including reduced blood loss, better vision, and improved instrumentation [12]. The first step in the robotic approach is access to the pleural space with a trocar or a minithoracotomy. Any issue at this initial step caused by adhesions can limit the benefits offered by this approach. Pleural adhesions can prevent the lung from collapsing, increasing the risk of lung injury from port placement or thoracic entry through the minithoracotomy [13]. Initial planning of port placement and minithoracotomy site can prevent conversion to sternotomy due to lung injury or dense adhesions that prevent adequate surgical exposure for robotic setup [8]. Identifying an area in the thoracic space that shows less adhesions and allows a robotic setup for adhesiolysis can make sure that the operation continues minimally invasively. A significant portion of sternotomy conversions are due to pulmonary adhesions, and a conversion rate of 17.6% was reported in a series of minimally invasive mitral valve surgery patients [14,15]. Adhesions may also prevent safe internal mammary artery harvest or MIDCAB [16,17]. The sliding test can increase the safety of the procedure and help identify more accessible regions during thoracic access, targeting port entry or minithoracotomy sites free of adhesions [18].

Adhesions are expected in a majority of patients with ipsilateral previous thoracotomy and previous pneumonia [19]. In our cohort, previous cardiothoracic surgery was strongly associated with adhesions and a positive sliding test. However, certain patients with adhesions had no previous cardiothoracic surgery, most of whom also had no history of pneumonia, tuberculosis, or thoracic radiotherapy. Adhesions in these patients could not have been anticipated from the history alone and the sliding test proved useful in identification of adhesion in all these cases. In reoperative patients, where adhesions are expected, a positive test was always confirmed and helped localize adhesion-free areas for port placement and minithoracotomy planning. In patients without previous surgery, a negative test reliably excluded adhesions. 24 h chest tube output was higher in our patients with adhesions. This did not translate to longer intensive care unit times. There were no pulmonary injuries or conversions to sternotomy. The sliding test is useful in operative planning and safe initiation of robotic cardiac procedures, supplementing the benefits offered by the robotic approach in terms of reduced trauma and bleeding.

The first port is blindly placed in robotic-assisted MIDCAB cases and may be prone to pulmonary injury [20]. This can be either of the three ports that will be used for the endoscopic camera or the robotic arms. The order of this port placement can be tailored to choose the site with no expected adhesions. In cases with expected adhesions in all three port sites, performing the minithoracotomy before port placement can increase the safety of the procedure without affecting the robotic setup. For right-sided procedures, the minithoracotomy site and port placement can be tailored to stay within an area without dense adhesions. While this can reduce the working space of the robotic instruments within the thorax, the risk of complications is decreased. Dynamic cameras and robotic instruments allow successful surgery within a narrow corridor. Patients with extensive adhesions can be managed with this approach. Patients with previous thoracotomies, chest drains, or pneumonia can be effectively managed with the help of the sliding test. After a safe setup, robotic arms can safely remove adhesions in the rest of the thoracic space, even in extensively adhesive patients.

The superficial position of the pleura in the thoracic space and the large change in acoustic impedance at the pleural layers make it easy to assess using ultrasound [5]. The anterior and lateral chest wall can be examined in the supine position in the operating room with a high-frequency linear probe (5–10 MHz). Without the need to assess deeper structures, higher frequency allows better resolution at the pleural layers [21]. The visceral and parietal pleura can be distinctly visualized as two separate, echogenic layers between the posterior surface of the ribs, and their displacement against each other during the respiratory cycle produce the sliding sign [21,22]. In our experience, assessing this movement under anesthesia instead of conscious breathing has the added benefit of removal of intercostal muscle movement with forced ventilation, which can make the assessment of the sliding movement more difficult and give rise to more false positives. An observational study on thoracotomy patients measured pleural motion and found a mean 1.55 cm excursion of pleural layers in the upper thoracic wall and 2.4 cm in the lower thoracic wall [9]. This is explained by the greater distance of the upper thorax from the diaphragm resulting in less pronounced forces and shorter longitudinal excursions than the visceral pleura in the lower thorax [6]. Thus, a cutoff of 1 cm in the upper thoracic sites and 2 cm in the rest of the target sites for a positive sliding test may be reasonable [23]. Performing the sliding-test with ultrasound takes short time, is easy to learn, uses no radiation and has no additional burden to the patient, and can be performed with an ultrasound device that is readily available in the operating room [20]. The limitation of this test is its operator dependency. This limitation can be alleviated with standardization of location and excursion distance [23]. Supervised training with correlation of intraoperative findings may be important for adoption of the test with comparably accuracy.

The patient with the false-negative sliding test was observed to have loose adhesions during the operation and not dense adhesions which are more frequently associated with postoperative bleeding in thoracic surgery [23]. Chronic obstructive pulmonary disease is a cause of reduced pleural sliding due to overinflation of the lungs and bullae and results in false positivity of the sliding test [24]. Obesity is another cause of false positivity with reduced ultrasound resolution due to excessive fatty tissue [6,23]. In our six patients with false-positive sliding tests, none had a history of chronic obstructive pulmonary disease, four had a body mass index > 30 and one had a body mass index of 27.5. Based on this observation from our cohort, obesity may contribute to false positivity of the sliding test in cardiac surgery patients. The pleural sliding should be interpreted accordingly in patients with elevated body mass index and a tailored, patient-specific approach to surgical planning and setup remains essential to mitigate risks and optimize outcomes.

The clinical cost of a false-positive test was limited, as its main consequence was a change in the order of port insertion: the first port was placed at a site without suspected adhesions, and the remaining ports were inserted under endoscopic vision. When all sites were positive, the minithoracotomy that is already planned for robotic-assisted MIDCAB is performed before port insertion. In valve procedures, the minithoracotomy was placed more anteriorly or laterally as guided by the test. In some cases, this resulted in a reduced working space, but the articulating instruments and three-dimensional endoscopic vision of the robotic system compensated for this.

In a meta-analysis of ten studies on thoracic surgery patients, the pooled sensitivity and specificity of preoperative lung ultrasound for pleural adhesions were 71% and 96%, respectively [23]. The specificity in our cohort is similar to this estimate, whereas the sensitivity is higher than reported in thoracic surgery studies. Direct comparison is limited by differences in the patient population and test conditions. Most thoracic surgery studies examined awake patients during voluntary breathing, whereas our test was performed after intubation under controlled positive-pressure ventilation, which provides uniform lung excursion without intercostal muscle activity [6,7].

Preoperative CT evaluation of pleural adhesions has been studied in thoracic surgery with limited accuracy for conventional CT and better results with dynamic, four-dimensional techniques [25]. Although all patients underwent preoperative contrast-enhanced three-dimensional CT scans, these scans did not provide precise information at the planned port sites and on the sliding motion of the pleura. A prospective study evaluating the incremental value of the sliding test over CT in robotic cardiac surgery would be a valuable next step.

This study was performed in a single center with high experience in robotic cardiac surgery, which may limit the generalizability of our findings. The small number of patients with adhesions limits the precision of the sensitivity estimate, and larger series are needed to confirm these findings. Ultrasound is an operator-dependent imaging modality. All examinations were performed by a single experienced surgeon, which ensured consistency within the study but did not allow assessment of inter-operator variability. Findings from different centers with varying patient characteristics can provide further information about the utility of the sliding test in robotic cardiac surgery.

## 5. Conclusions

Pleural adhesions are not rare in patients undergoing robotic cardiac surgery and their optimal management enhances the safety and efficiency of robotic cardiac procedures, particularly in patients with a history of thoracic interventions. By guiding tailored modifications to the robotic setup based on the location of adhesions, the pleural sliding test reduces the risk of pulmonary injury during thoracic access.

## Figures and Tables

**Table 1:** Baseline characteristics of study patients.

	*n* = 300
Body Mass Index	28.0 ± 4.7
History of Smoking	172 (57.3%)
Hyperlipidemia	153 (51.0%)
Chronic Obstructive Pulmonary Disease	7 (2.3%)
Previous Cardiothoracic Surgery	16 (5.3%)
Sternotomy	12 (4.0%)
Ipsilateral Thoracotomy	5 (1.7%)
History of Pneumonia	6 (2.0%)
History of Pulmonary Tuberculosis	3 (1.0%)
Prior Thoracic Radiotherapy	5 (1.7%)

**Table 2:** Type of robotic cardiac operation.

	*n* = 300
Robotic-assisted MIDCAB	163 (54.3%)
Mitral Valve Surgery	
Mitral Repair	58 (19.3%)
Mitral Repair + CABG	1 (0.3%)
Mitral Repair + Myectomy	1 (0.3%)
Mitral Valve Replacement	20 (6.7%)
Tricuspid Valve Surgery	
Tricuspid Valve Repair	2 (0.7%)
Tricuspid Valve Replacement	6 (2.0%)
Aortic Valve Surgery	
Aortic Valve Replacement	1 (0.3%)
Mitral and Tricuspid Valve Surgery	
Mitral and Tricuspid Repair	13 (4.3%)
Mitral Replacement and Tricuspid Repair	11 (3.7%)
Mitral Repair and Tricuspid Replacement	1 (0.3%)
Mitral and Tricuspid Replacement	2 (0.7%)
Aortic and Mitral Valve Surgery	
Aortic and Mitral Valve Replacement	1 (0.3%)
Aortic, Mitral and Tricuspid Valve Surgery	
Aortic and Mitral Replacement and Tricuspid Repair	1 (0.3%)
Other	
Atrial Septal Defect Repair	10 (3.3%)
Pulmonary Venous Return Anomaly Repair	2 (0.7%)
Atrial thrombus excision	2 (0.7%)
Pericardial Window	1 (0.3%)
Myxoma	4 (1.3%)

CABG: Coronary Artery Bypass Graft; MIDCAB: minimally invasive direct coronary artery bypass.

**Table 3:** Baseline characteristics and operative outcomes of patients with and without adhesions.

	Without Adhesions (*n* = 280)	With Adhesions (*n* = 20)	*p*
Age	59.9 ± 13.5	66.2 ± 14.6	0.059
Female Gender	85 (30.4%)	6 (30.0%)	0.973
Hypertension	138 (49.3%)	8 (40.0%)	0.422
Diabetes Mellitus	98 (35.0%)	3 (15.0%)	0.067
Body Mass Index	28.0 ± 4.4	27.9 ± 7.4	0.491
Hyperlipidemia	146 (52.1%)	7 (35.0%)	0.138
Smoking	163 (58.2%)	9 (45.0%)	0.248
Chronic Obstructive Pulmonary Disease	6 (2.1%)	1 (5.0%)	0.386
History of Pneumonia	4 (1.4%)	2 (10.0%)	0.054
History of Pulmonary Tuberculosis	2 (0.7%)	1 (5.0%)	0.188
Prior Thoracic Radiotherapy	4 (1.4%)	1 (5.0%)	0.294
Previous cardiac or ipsilateral thoracic surgery	3 (1.1%)	13 (65.0%)	<0.001
CPB Time	134.9 ± 47.3	149.4 ± 55.1	0.254
CC Time	65.5 ± 24.6	52.3 ± 36.5	0.349
Mechanical Ventilation (hours)	6.1 ± 15.4	8.1 ± 8.4	0.148
ICU stay (h)	24.2 ± 22.3	33.9 ± 24.9	0.102
24 h chest tube output (mL)	533.7 ± 426.5	1100.2 ± 1025.4	0.006
Blood transfusion	19 (6.8%)	6 (30.0%)	0.003
Pulmonary injury	0 (0%)	0 (0%)	
Reoperation for bleeding	0 (0%)	1 (5.0%)	0.067
Conversion to sternotomy	0 (0%)	0 (0%)	

CPB: cardiopulmonary bypass; CC: cross clamp; ICU: intensive care onit.

**Table 4:** The predictive ability of the sliding test.

	Intraoperative Adhesions	Intraoperative No Adhesions	Total	Predictive Value
Sliding Test Positive	19	6	25	PPV 76.0% (54.9–90.6%)
Sliding Test Negative	1	274	275	NPV 99.6% (98.0–100.0%)
Total	20	280		
	Sensitivity	Specificity		
	95.0% (75.1–99.9%)	97.9% (95.4–99.2%)		

NPV: negative predictive value; PPV: positive predictive value.

**Table 5:** The predictive ability of the sliding test for preoperative surgery.

	Previous Surgery (*n* = 16)	No Previous Surgery (*n* = 284)
Adhesions	13 (81.3%)	7 (2.5%)
Sensitivity	92.3% (64.0–99.8)	100% (59.0–100.0)
Specificity	100% (29.2–100.0)	97.8% (95.3–99.2)
Positive Predictive Value	100% (73.5–100.0)	53.8% (25.1–80.8)
Negative Predictive Value	75.0% (19.4–99.4)	100% (98.6–100.0)

## Data Availability

The data supporting the findings of this study are available from the corresponding author upon request.

## Supplementary Materials

The following supporting information can be downloaded at https://www.mdpi.com/article/10.3390/jcdd13100495/s1, Video S1: Sliding test performed in the operating room.

## Abbreviation

The following abbreviation is used in this manuscript:

MIDCAB	minimally invasive direct coronary artery bypass
